# A mixed-methods evaluation of the impact of a person-centered family planning intervention for community health workers on family planning outcomes in India

**DOI:** 10.1186/s12913-020-05995-9

**Published:** 2020-12-11

**Authors:** Nadia Diamond-Smith, Claire McDonell, Ananta Basudev Sahu, Kali Prasad Roy, Katie Giessler

**Affiliations:** 1 Department of Epidemiology and Biostatistics, Institute for Global Health Sciences, University of California, 550 16th Street, 3rd Floor, San Francisco, CA 94158 USA; 2 Population Services International, C-445, Bipin Chandra Pal Marg, Block C, Chittaranjan, New Delhi, Delhi, 110019 India

**Keywords:** Person-centered care, Family planning, India, Community health workers

## Abstract

**Background:**

Person-centered quality for family planning has been gaining increased attention, yet few interventions have focused on this, or measured associations between person-centered quality for family planning and family planning outcomes (uptake, continuation, etc.). In India, the first point of contact for family planning is often the community health care worker, in this case, Accredited Social Health Activists (ASHAs).

**Methods:**

In this study, we evaluate a training on person-centered family planning as an add-on to a training on family planning provision for urban ASHAs in Varanasi, India in 2019 using mixed methods. We first validate a scale to measure person-centered family planning in a community health worker population and find it to be valid. Higher person-centered family planning scores are associated with family planning uptake.

**Results:**

Comparing women who saw intervention compared to control ASHAs, we find that the intervention had no impact on overall person-centered family planning scores. Women in the intervention arm were more likely to report that their ASHA had a strong preference about what method they choose, suggesting that the training increased provider pressure. However, qualitative interviews with ASHAs suggest that they value person-centered care for their interactions and absorbed the messages from the intervention.

**Conclusions:**

More research is needed on how to intervene to change behaviors related to person-centered family planning.

**Trial registration:**

This study received IRB approval from the University of California, San Francisco (IRB # 15–25,950) and was retrospectively registered at clinicaltrials.gov (NCT04206527).

**Supplementary Information:**

The online version contains supplementary material available at 10.1186/s12913-020-05995-9.

## Background

Ensuring that women are supported in making informed choices about family planning methods and are treated in a respectful, autonomous and communicative (or “person-centered”) manner, is essential in all settings, including in India, which has a history of coercive family planning programs [1]. In 2016, 54% of women in India use a method of family planning (48% a modern method), primarily female sterilization (36%), condom (5.6%) and Pills (4.1%) [2]. The fist point of contact for many women and men about family planning in India is their local community health worker—a cadre of health workers that receives little attention (training, support, intervention) yet carries much of the burden of face-to-face care provision. Across India, reproductive healthcare services such as access to family planning and facility-based delivery services are provided free of charge by the National government to facilitate efforts to improve maternal and neonatal health outcomes [3]. Despite these efforts, 20% of women in India have an unmet need for family planning, which can be linked to high rates of maternal mortality and incidence of unsafe abortions [4, 5]. Even with substantial financial investments put forth by the government, the annual increase of modern family planning use between 2012 and 2018 among reproductive aged women in India has only been 0.1% [6].

In 2005, the Government of India launched the National Rural Health Mission (NRHM) with the objective to bring quality and affordable health care, including access to family planning, to communities across the country in rural settings [7]. Efforts to improve quality and access by the NRHM included the deployment of Accredited Social Health Activists (ASHAs), who are female community health workers stationed directly within communities that act as the initial contact with the formalized healthcare system, focusing on maternal and child health and family planning. In general, ASHAs are female residents of the community they serve, between 25 and 45 years old, and have a formal education up to class 8 [8]. In 2013, the NRHM evolved into the National Health Mission (NHM) and grew to include the National Urban Health Mission. This expansion aimed to meet the unique needs of urban slum populations [9]. The Government of India cited “overcrowding” of clinics and inefficient outreach and referral systems as barriers that make urban slum populations especially vulnerable to exclusion from the health system [10, 11]. Each urban public health center (UPHC) caters to a slum population of between 25,000–30,000 individuals [10]. As per the government guidelines, there is an ASHA for every 1000–2500 people in urban areas and they can cover between 200 and 500 households each [10].

As of 2015, there was approximately one ASHA for every 1000 people across the nation [12]. This spread puts ASHAs in a unique position to extend the availability of family planning services to hard-to-reach communities, rural and urban alike. Even with a large workforce focused on family planning, 20% of women have an unmet need [13]. Recent reviews of Indian nation health policy found that quality of care is a key concern in Indian public health service delivery, and is potentially contributing to this gap [14].

In response to this significant access challenge, the Government of India has committed to deliver “quality assured [family planning] services to the hardest-to-reach in rural and urban areas” by 2020 [15]. In line with this goal, quality improvement and monitoring systems have been integrated into many health programs including the NHM, as well as the Reproductive, Maternal, Newborn, Child Health and Adolescent (RMNCH+A) program, launched in 2013 [14]. These quality improvement initiatives do not however regularly evaluate “patient satisfaction” nor the quality of interactions between patients and providers [14]. Person-centeredness is also left out of these quality initiatives. By “person-centeredness” we mean care that addresses domains of quality related to respect, communication, trust, etc., and that is focused around meeting the needs and desires of the person receiving the care [16, 17]. Person-centered care (PCC) is grounded in the demand side, rather than the supply side (contraceptive methods, facilities, provider technical skills training/knowledge) side of contraceptive care. A review of literature has found that interventions focused on PCC dimensions in family planning were associated with patient satisfaction, increased knowledge, with limited and mixed results about method uptake and sustained use [16]. Dimensions of quality included in these assessments were related to communication, privacy/confidentiality, supportive care, dignity, autonomy social support and trust.

As part of a larger project on person-centered care for family planning, delivery and abortion, we developed and validated a scale to measure person-centered family planning (PCFP) in India (and Kenya), described in more detail elsewhere [17]. The final validated scale in India included 22 items that fell into two domains: “autonomy, respectful care, and communication” and “health facility environment.” The scale was validated in the same state of India (Uttar Pradesh) as the current study, however, it was validated in a population of women seeking family planning services at a facility and being seen by a trained, professional health care provider. It has not yet been validated among women interacting with a community health worker (CHW). There is no known measure of person-centered interactions for CHWs, who provide a large proportion of care for women, especially for family planning, globally.

Since ASHAs are the first point of contact about family planning for many women in India, we developed a training for ASHAs (described in more detail below) on PCFP to be added onto a training on family planning quality more broadly. To evaluate this intervention, we use mixed methods comprised of [1] surveys with women seen by ASHAs that did and did not receive this additional training (“intervention”) and [2] qualitative interviews with ASHAs who received the intervention. We conducted this study in Varanasi, Uttar Pradesh, which has a total population of 36.77 lakh (approximately 3.7 million) per the 2011 Indian census. Of the total population, 44.4% live in urban areas. About 57% of currently married women in the age group of 15–49 years were using any family method in the urban area of Varanasi district, where our study took place [2].

Our first aim was to test if additional PCFP training given to ASHAs was associated with changes in person-centered quality for family planning scores by comparing scores among women who have worked with trained ASHAs versus those women that have worked with non- trained ASHAs. Additionally, we assessed whether person-centered quality for family planning scores were associated with a higher likelihood of family planning method adoption, continuation, and other markers of quality. By integrating the voices of the ASHAs through qualitative interviews, we are able to explore more in depth why certain domains of PCFP may have been more or less salient for ASHAs.

### Intervention

The PCFP intervention built upon an ongoing effort by the government of Uttar Pradesh, India to improve family planning access through the deployment of ASHAs into urban areas of Uttar Pradesh. The intervention was conducted within the context of The Challenge Initiative for Healthy Cities (TCIHC) program, which works alongside the government to provide enhanced training in family planning counselling and method options to this new cadre of urban ASHAs. The aim of TCIHC is to encourage uptake of modern family planning methods for delay of first pregnancy and/or spacing between births among urban women with unmet need aged 18 to 24.

The intervention described in this paper was added to a standard in-service family planning training focused on defining family planning, risks and benefits associated with modern family planning methods, clarification of temporary versus permanent methods of family planning and how each can be used, efficacy of methods, communication and counseling techniques, and the role and duties of the ASHA within the government family planning program. In the intervention arm, ASHAs received an additional training module focused on salient PCFP domains as described by Sudhinaraset and colleagues (Sudhinaraset et al., 2018) [17]. The intervention consisted of a four-hour training focused on areas of PCFP that may be most relevant to community health workers; namely respect, communication, trust, and autonomy. The training also covered the importance of person-centered care, family planning method mix and supporting clients (women) in choosing appropriate family planning methods for themselves (informed choice). The training was interactive, including case studies and role play sessions for the ASHAs to practice providing counselling to different types of clients and to think through their own experiences of poor treatment, discrimination, and their own unconscious bias. The comparison group of ASHAs received the same standardized in-service family planning training as those in the intervention arm and did not receive additional training in PCFP principles.

The intervention training was initially pilot tested via a training of trainers (ToT) in Uttar Pradesh and conducted with program managers, clinicians and project officers with expertise in family planning. ToT participants provided feedback on cultural acceptability and appropriateness, as well as relevance of PCFP focus areas for community health workers. The intervention training was adapted accordingly and additionally pilot-tested with a group of 21 ASHAs working in an urban area comparable to study site locations. Pre-post pilot survey results indicated that ASHAs agreed or strongly agreed that the training was helpful to their work, the training content was important for ASHAs, training in PCFP would help them to provide better care, and that they desired further training in PCFP. Further adaptations to the intervention training content were then made based on feedback received from pilot participants during the training and within opened ended responses contained in the pre-post survey.

The actual intervention (training) was conducted with two different groups of 20 urban ASHAs each in January 2019. Pre-post survey responses indicated that almost all (32/40) training participants agreed that the training in PCFP would help them to provide better care and more than two-thirds agreed that they learned something new during the intervention training.

## Methods

### Evaluation

We evaluated the additional PCFP component add-on to the family planning training provided through TCIHC in four intervention UPHCs compared to four control UPHCs in Varanasi, Uttar Pradesh. Intervention and control sites were matched by considering the estimated number of women with unmet family planning need, number of ASHAs, socio-demographics of the population in each UPHC”, urban population of women, and age ratios.

We conducted surveys with women who had been visited by an ASHA in both control and intervention areas approximately 3 months post-intervention. In order to detect a 10% difference in mean PCC score between intervention and control groups, we interviewed 542 women per arm, for a total of 1084 women. Within each arm, we interviewed 271 women who had taken up family planning and 271 women who had not. Systematic random sampling was used to draw sample respondents from a register kept by ASHAs for their specific supervision geographies of users and non-users of family planning. Names and status of method use were then cross-checked with UPHC health records that indicated which women were or were not using family planning methods or had discontinued or switched their family planning method. Survey data were collected between April–June of 2019. We also conducted qualitative interviews with a subset of ASHAs in both intervention and control areas (*N* = 20) in April and May of 2019. Only the results of interviews with intervention ASHAs are described in this paper.

Surveys with women: Women in both control and interventions areas who had seen an ASHA within the previous 3 months were surveyed to understand the quality of their experience with the ASHA and whether the woman had taken up a family planning method of her choice following interacting with the ASHA. Eligibility criteria for the survey included women in the age group of 15–49 years who had been seen by an ASHA from one of the four intervention or four control UPHCs in the last 3 months. Women were identified through the ASHA registers. Since we were concerned that family planning uptake would be low, we designed our study to ensure that half of respondents would be family planning users or adopters. Of the total sample (*N* = 1084), 369 women had adopted a new method, 172 had switched methods, and the remaining 542 women had not adopted a method since meeting with the ASHA at a minimum of 3 months prior to study participation.

To recruit potential survey participants, a female enumerator trained in quantitative data collection read an introductory script outlining the length of the survey and confidentiality of responses to women who were contacted in their homes. To protect the privacy of potential respondents, the script did not indicate specifically that the survey was focused on experiences with family planning services, rather, that the survey was focused on general healthcare services received via the ASHA. If a woman agreed to participate and/or was interested in learning more about the survey, a consent form was read to and/or read by the participant which outlined voluntariness of participation, efforts that would be undertaken to protect privacy of responses and data from anyone outside of the research team inclusive of ASHAs, healthcare providers or health facilities, clarification that the research team was not affiliated with any health facility, and that participants were free to withdraw at any point. The verbal consent form explicitly stated that the questions in the survey were focused on family planning. Women who agreed to participate in the study provided verbal consent and were asked if they would prefer to conduct the survey within their own homes or at an outside location such as a community center. The enumerator then confirmed whether the participant wanted to conduct the survey at the time of consent or schedule participation at a more convenient time. The survey was a standardized structured questionnaire that took approximately 30–40 min to conduct. The majority of women chose to be interviewed in their homes; very few opted to be interviewed at a local community center. To ensure additional privacy for women that were interviewed in their homes, the female enumerator requested that the woman find a secluded place to participate in the interview. Additionally, the enumerator requested that additional people within the home, including husbands and mothers-in-law, allow the woman to participate in the survey in a private place within the home. Women who did not meet eligibility criteria, refused participation following an explanation of the study’s purpose or who refused to consent to participation were excluded from the study.

Qualitative interviews with ASHAs: A sample of twenty ASHAs was purposively selected from the intervention and control arm of the study for interviews lasting 1 to 2 h. ASHA were sampled to be roughly half in control and half in intervention groups and within each of those, we purposively sample by length of time working as an ASHA (working for < 3 years and more than 3 years). In-depth interview guides were developed to elicit the perspectives of ASHAs on their experiences providing family planning counseling to clients. Intervention participants were also asked about their perception of the integration of PCFP into their existing family planning practices. Before starting each interview, verbal informed consent was collected from participants by the lead interviewer. Participants were also informed that involvement in the interview was voluntary and that they were free to terminate participation at any point. Using an introductory script, participants were also informed that no information from the voluntary interview would be shared with their supervisors, clinic staff, or any government officials in a way that could identify them. Interviews were audio recorded and notes were taken throughout their duration. Audio recordings were transcribed in Hindi and then translated into English for analysis. For the purposes of this paper we only discuss findings from the intervention ASHAs.

This study received IRB approval from the University of California, San Francisco (IRB # 15–25,950).

### Analysis

#### Quantitative measures

Person-centered care: There were 15 individual items asked to women about their person-centered care experience with the ASHA. We adapted the PCFP scale discussed above that was validated in Uttar Pradesh for women who sought care in a facility to be more appropriate for women seeing an ASHA [17]. Some items were dropped that specifically related to more technical procedures or facility environment, leaving 15 of the original 22 items. Remaining items were slightly re-worded to be reflective of visits in the home with an ASHA.

Our first step was to validate the PCFP scale previously validated in India among women who saw a provider in a facility among women who saw the ASHA. The original PCFP scale included the following items: the provider introducing themselves, being treated with respect, trusting the provider, being given the “best care”, given information, being involved in decisions, having things explained to them, understanding what was happening, being involved in the family planning method choice, being allowed to ask questions, being allowed to have someone stay with them in their visit, and feeling that their fears were supported (Table 2) [17]. We followed the same factor analysis procedures as in the initial validation, described in detail in Sudhinaraset et al. [17] The initial validation paper identified two sub-scales. We only included items from the “autonomy, respectful care, and communication” sub-scale because the other sub-scale was related to the health facility environment which was not relevant for community health workers visiting women in their homes. We found that all of the items in the PCFP sub-scale loaded well onto 1 factor in this analysis (alpha = 0.939). All items included in the original PCFP scale used in this analysis.

We thus created a summary score that ranged from 0 to 43, with higher scores meaning that the woman had an overall more positive, person-centered experience. We wanted to also explore each item individually. Each item was ranked on a 4-point scale (“none of the time”, “some of the time”, “most of the time” and “all of the time”, for most indicators). To make interpretation and analysis easier, we created a binary value for each item where the lowest two response categories were grouped and the highest two grouped.

Other indicators of person-centered interactions: To better understand how our measure of PCFP is associated with other commonly used measures, we looked at two other measure of interactions between clients and providers. The first is a question that asked if the woman felt the ASHA was involved too much, too little, or the right amount in the decisions about what method to choose. This was made into a binary variable of “too much/too little” compared to the right amount. We determined that anything other than “the right amount” as being indicative of poorer quality care as it did not meet the needs of the woman herself. The second indicator was a question asking the women if she felt that the ASHA had a preference about what method she choose: Extremely strong preference, strong, moderate, slight, none. A binary was created of extremely strong and strong compared to all others, with the interpretation that strong preferences were an indicator of pressure.

Family Planning use: The primary outcome variable was family planning uptake at 3 months post-ASHA training. This was measured by a question that women answered asking if she had adopted a family planning after meeting with an ASHA within the previous 3 months, or if she switched to a new method since the ASHA’s visit.

Socio-demographic control variables: We controlled for a number of socio-demographic factors which could impact women’s family planning use and person-centered experiences, based on previous studies in this setting. We controlled for age in groups (18–24, 25–29, 30–34 and over 35), education in groups (Illiterate/No school/Primary, Post-primary/vocational/Secondary, college or above, and still in school), and occupation (being a homemaker or not). We also controlled for caste groups (Scheduled caste/tribe (lowest), Other Backwards Castes, and General Caste) and religion (Muslim vs. Hindu). Finally, we controlled for if the woman stated that she desired more children, as this is important for understand family planning uptake.

Quantitative Analysis: First, we show the socio-demographic characteristic of women in the intervention and control groups, and overall, including testing for significant differences, using percentages and chi-squared tests. Next, we explored whether person-centered care scores or individual items (as binary values) differed between intervention and control participants, using means, percentages, and t-tests. We then ran multi-variable regression models, controlling for the socio-demographics described above, to explore the association between being in the intervention and the full PCC score. Next, we explored the association, using multi-variable logistic regression models, between PCC-scores and family planning uptake, controlling for the same socio-demographic variables. All analyses were run using STATA version 15 [18].

Qualitative Analysis: Initial summary memos were drafted for each interview transcript and continuously refined throughout the data analysis process. Each interview transcript then went through a multi-phase iterative coding process using ATLAS.ti version 8.4.2 [19]. .The coding process involved cycles of open coding, axial coding, and selective coding. A codebook was developed and continuously refined until agreed upon by three researchers (NDS, KG, CM). Any additions or changes to the codebook were documented. In addition to a codebook, a data matrix was created to visualize emerging themes and refine potential theories. Data were analysed using Grounded Theory and analysis continued until thematic saturation was deemed to be reached [20].

## Results

Description of study population: Most women were 25–29 year old (38.4%), with 30.5% being 18–24, 18.5% being 30–34 and 12.5% over 35 years (Table 1). Most women were illiterate or had none or primary school (40.1%), 19.3% had post-primary/ vocational/ secondary school, 33.1% college or above and 7.5% were still in school. Most were homemakers (93.8%). Most were other backwards caste (64.5%) or Scheduled caste/Scheduled tribe (21.8%) and were Hindu (81.8%). Just under half wanted more children (46.7%). There were significant differences between intervention and control groups, with the intervention group being slightly older, less educated, more other backwards caste/less general caste, more Muslim, having more sons and not desiring additional children. Just over 49% of women had adopted a method at 3 months post-ASHA visit, with no difference between intervention and control groups.

**Table 1:** Demographics of the control and intervention survey participants (family planning clients), N, %

	Intervention		Control		Total	
	No.	%	No.	%	No.	%
Age group*						
18–24	154	28.4	177	32.7	331	30.5
25–29	189	34.9	227	41.9	416	38.4
30–34	102	18.8	99	18.3	201	18.5
35 and over	97	17.9	39	7.2	136	12.5
Education*						
Illiterate/No school/Primary	236	43.5	199	36.7	435	40.1
Post-primary/vocational/Secondary	88	16.2	121	22.3	209	19.3
College or above	171	31.5	188	34.7	359	33.1
still in school	47	8.7	34	6.3	81	7.5
Occupation						
Working	29	5.4	38	7	67	6.2
Homemaker	513	94.6	504	93	1017.00	93.8
Caste group*						
SC/ST	99	18.3	137	25.3	236	21.8
Other Backwards Castes	381	70.3	317	58.6	698	64.5
General	62	11.4	87	16.1	149	13.8
What is your religion*						
Hindu	388	71.6	498	92.1	886	81.8
Muslim	154	28.4	43	7.9	197	18.2
Desire More Children*						
No	310	58.5	257	48.2	567	53.3
Yes	220	41.5	276	51.8	496	46.7
Adopted a method at 3 months						
No	335	51	292	50.2	627	50.6
Yes	322	49	290	49.8	612	49.4

*significant at the *p* < 0.05 level difference between control and intervention

### Quantitative evaluation findings from the survey with clients

The overall PCC score was not significantly different between the intervention and control groups, with a mean of about 29.3 (range from 0 to 43) (Table 2). Women in the control arm in general rated individual PCFP items slightly lower, although this difference was only significant for 4 items: the ASHA introducing herself, showing respect, feeling the ASHA wanted the best for her and being allowed a person of her choice to stay during the visit.

**Table 2:** Differences between women who saw intervention and control ASHAs in percent who report each person-centered care items, percentages shown unless otherwise stated

	Intervention, percent of women reporting the two highest responses^a^		Control, percent of women reporting the two highest responses^a^	
	N	%	N	%
Total	536	100	541	100
PCC score (mean, IQR)	29.30	(28,36)	29.19	(27,35)
ASHA introduced herself when ASHA came (*p* = 0.0000)	517	96.5	470	86.9
ASHA treated her with respect (*p* = 0.0000)	520	97	481	88.9
ASHA wanted the best for her (*p* = 0.0464)	468	87.3	449	83
Given enough information about her care in order to feel like she understood what was happening	423	78.9	420	77.6
ASHA involved her in decisions	410	76.5	409	75.6
ASHA clearly explained things	436	81.3	442	81.7
ASHA answered in a way that she could understand when she had questions	450	84	450	83.2
ASHA supported her anxieties and fears about family planning procedure or method choice	380	70.9	398	73.6
Felt she could ask the ASHA any questions they had	464	86.6	451	83.4
Felt she was allowed to have someone she wanted to stay with her during the visit (*p* = 0.0366)	390	72.8	362	66.9
Felt the ASHA was available when she want to speak to the ASHA, had questions, or needed support	442	82.5	434	80.2
Felt the ASHA took the best care of her	400	74.6	415	76.7
Felt the ASHA cared about her as a person	447	83.4	454	83.9
Had complete trust in the ASHA with regards to her care	442	82.5	433	80

^a^Two highest = “most of the time” or “all of the time” compared to “none of the time” or “some of the time”,

The majority of women reported that their ASHA was involved exactly the right amount in their family planning method choice (72.9%), with 16% stating they wished she was involved less and 11% that she was involved more (Table 3). Few differences emerged between the intervention and control. About 30% of women overall said that their ASHA had no preference or a slight preference, and only 10.6% that she had an extremely strong preference. It appears that women in the intervention group reported slightly higher levels of preference than women in the control group.

**Table 3:** Distribution of responses to other two quality measures by intervention and control groups, N(%), column percentages

	Intervention		Control		Total	
	No.	%	No.	%	No.	%
How do you feel about how involved your ASHA was with helping you choose a family planning method?						
I wish my ASHA had been less involved	39	15.4	40	16.5	79	16
My ASHA was involved exactly the right amount	183	72.3	178	73.6	361	72.9
I wish my ASHA had been more involved	31	12.3	24	9.9	55	11.1
Did your ASHA have a preference for what family planning method you should use?						
No preference	108	20	129	23.8	237	21.9
Slight preference	47	8.7	65	12	112	10.4
Moderate preference	91	16.8	120	22.2	211	19.5
Strong preference	232	42.9	174	32.2	406	37.5
Extremely strong preference	63	11.6	52	9.6	115	10.6
Don’t know	0	0	1	0.2	1	0.1

Table 4 shows that women who had higher person-centered family planning scores (rated their interaction as better) for their interaction with the ASHA had increased odds of taking up a family planning method (OR = 1.04***, *p* = 0.000). Receiving care from an intervention ASHA was not associated with PCFP scores. Receiving care from an intervention ASHA was also not associated with saying that the ASHA was involved the “right amount.” However, receiving care from an intervention ASHA was associated with increased odds of a woman saying that the ASHA had a “strong” or “extremely strong” preference for what method she chose (OR = 1.861,*p* = 0.000). Age was significantly associated with outcomes in all models, other control variables were not consistently associated.

**Table 4:** Association between PCFP score and current family planning use (at 3 month follow up), and the impact of the intervention on person-centered related outcomes

	Currently using family planning (at 3 month follow up) (Odds ratio, standard errors)	PCFP Score (coefficient, standard errors)	ASHA had a strong or extremely strong preference about Method (Odds ratio, standard errors).	ASHA was Involved the right amount (Odds ratio, standard errors).
PCFP score	1.041*** (0.00748)			
Intervention		0.876 (0.528)	1.861*** (0.249)	1.098 (0.152)
Age (compared to 18–24)				
25–29	0.911 (0.143)	0.249** (0.176)	1.234 (0.192)	0.900 (0.145)
30–34	1.093 (0.211)	0.163** (0.142)	1.146 (0.219)	0.912 (0.180)
Over 35	0.468*** (0.111)	0.0299*** (0.0312)	0.466*** (0.110)	0.484*** (0.126)
Education (compared to illiterate/none/primary				
Secondary/post-secondary	1.148 (0.204)	0.452 (0.361)	1.287 (0.227)	0.702* (0.129)
College	0.998 (0.153)	0.0457*** (0.0312)	1.246 (0.189)	0.601*** (0.0964)
Still in school	1.001 (0.255)	4.087 (4.712)	1.235 (0.313)	0.975 (0.251)
Occupation (homemaker compared to working)	0.648 (0.176)	3.062 (3.693)	1.303 (0.354)	1.103 (0.326)
Caste (compared to Scheduled Caste/tribe)				
Other Backwards Caste	1.178 (0.192)	3.356* (2.465)	0.898 (0.146)	1.298 (0.224)
General	1.154 (0.258)	4.657 (4.667)	0.981 (0.217)	1.437 (0.337)
Religion (Muslim vs Hindu)	0.907 (0.159)	0.460 (0.370)	1.017 (0.180)	0.896 (0.167)
Desire More Children	1.123 (0.153)	0.196*** (0.120)	0.945 (0.127)	1.027 (0.144)
Constant	0.479* (0.210)	4.476e+ 13*** (7.532e+ 13)	0.501* (0.189)	0.582 (0.235)
Observations	1056	1056	1056	1056
R-squared		0.048		

*** *p* < 0.01, ** *p* < 0.05, * *p* < 0.1

### ASHA’s perspectives on the PCFP training from the qualitative interviews

The qualitative sample (*N* = 20) included 11 intervention ASHAs who had participated in the PCFP training and nine control ASHAs who did not. Respondent ages ranged from 28 to 42 years (mean: 34.8). One half of the sample had completed lower secondary school up to grade ten and the other half had completed upper secondary school up to grade 12. All quotes are from the Intervention ASHAs, as they were the only ones who were asked specific questions related to the PCFP training.

ASHAs already had deeply engrained PCFP values, including respect, support, communication, and maintaining privacy. Despite this, the ASHAs still felt that there was value in the PCFP training, and described how it changed their perspective or practice related to various domains of PCFP.

One ASHA noted that the training changed the way she thought about her role as an ASHA. Afterwards, the ASHA not only started viewing herself as an agent of change, but also recognized that using disrespectful treatment can impact a beneficiary’s choice to pursue family planning care. In her interview she shared what she and some of her fellow ASHAs garnered from the training: *“We have to first change our behavior (before) we can change others’. This is what we found different. Suppose if someone (behaved) badly with me, if (I) would have also done the same, then they wouldn’t have called us back.”* (Respondent 4, Intervention).

Communication was another main topic in the PCFP training. One respondent shared how the PCFP training changed her perspective on respectful communication:

*I would get angry before, not now. I tell them, “Don’t get anything done, at least you can talk with me. If you are busy now, I shall come after an hour and talk to you.” When I talk to them softly, they understand me. And if she is busy, she will not listen to me. I should talk with her later. Then she will think about what I said. I should talk with the beneficiary according to her convenience.* (Respondent 16, Intervention)

Respondents directly and indirectly spoke about elements of effective communication throughout their interviews. ASHAs noted that when providing care, it was important to communicate in a way that beneficiaries will understand. As one ASHA shared about applying clear communication to family planning counseling: *“We have to explain all thing(s) about family planning, in their language. If we explain (to) them in theoretical language, then they will understand nothing.”* (Respondent 5, Intervention).

In the PCFP training, ASHAs learned about respecting autonomy when meeting with beneficiaries. One respondent reflected on PCFP teachings: *“We should listen to them [beneficiaries]. We should not impose our choice on them. We should not talk with them in harsh manner; not be angry with them. I should not say, ‘Get Multiload (IUD) inserted.’ This is imposing. I should ask her, “What is (your) choice?”* (Respondent 1, Intervention). A second respondent reiterated this and went on to specify how she applies PCFP components like respect and autonomy when interacting with beneficiaries: **“***Suppose if they’re not ready to use methods like sterilization or IUCD for whatever maybe the reason. [I] have to try to understand their problem. I cannot force them to use such methods. We cannot pressure them.”* (Respondent 5, Intervention).

Another respondent reflected on what she learned about transparency at the PCFP training: *“I got to learn that we should tell both good and bad things to the beneficiaries. We should tell all the products of family planning and let her choose. We cannot force them.”* (Respondent 13, Intervention).

Another ASHA reflected on applying the PCFP dimension ‘privacy’ in the home setting to help create space for beneficiary-led decision-making. She found confidentiality and privacy to the most important aspects of PCFP training:

*Most important of all was keeping everything confidential. Suppose we have visited…a (beneficiary) and everyone in her family is sitting nearby. Suppose I need to ask her about the Multiload (IUD), however other family members don’t know about her thinking of getting Multiload(IUD) done. Therefore, confidentiality becomes important here, so we will take her aside and discuss in private.* (Respondent 2, Intervention)

Another ASHA talked about how the PCFP training directly impacted her privacy practices and changed her strategy for speaking with women about family planning: *“We have to talk with her [the beneficiary] separately so that no one knows about it – secrecy. Before (the PCFP training) we started talking (with) others, so even if she wanted to take benefit, she could not.”* (Respondent 1, Intervention).

## Discussion

Improving the person-centeredness of interactions for all kinds of care, including reproductive health care, is important, from a human rights and health care perspective. Evidence of the impact on health outcomes helps bolster the case for person-centered approaches. Our findings add to limited previous research that person-centered care is associated with family planning outcomes, namely, family planning uptake and continuation [16]. This confirms that efforts to improve women’s experience receiving family planning are likely important not only for the experience itself, but actually lead to health behavior change. The next step—figuring out how to actually improve person-centered quality—may be more challenging, as we discuss below.

This evaluation did not find an impact of the add-on person-centered quality module to the family planning training offered by TCIHC on women’s overall PCFP scores. A few items in the PCPC scale were significantly associated with the intervention, including the ASHA introducing herself, and treating the respondent with respect. There are several possible explanations for this finding, the first of which is simply that a short training such as this is not effective for behavior change among community health workers and interventions that are longer or include multiple sessions over time, are integrated into initial training, or target system level cultural change may be more appropriate.

Another set of explanations revolved around the items asked themselves. The items in the PCFP scale were validated in Uttar Pradesh, but in a different population (women seeking care in facilities) [17]. Although we adapted the items and removed those we felt were not relevant, it is possible that different types of questions or topics would be more relevant to the ASHA-client interaction. The items held together in the factor analysis and loaded together in the same way, however, these might not be the most salient to women interacting with an ASHA. Conducting cognitive interviews to test these adapted items and exploring other possible domains that should have been covered would have helped to ascertain if this was the explanation.

A related explanation is that the items that were significantly associated with the intervention, most specifically respect, are actually a better summary indicator of what we define as PCFP than the entire list of indicators included. In fact, many other scholars who are researching “person-centered” care call this same construct “respectful care”, especially in the maternity literature, although increasingly in the family planning literature as well [21–24]. Perhaps a simpler and more direct approach to obtain the same information about women’s experiences could be to ask this singular question, as it encompasses multiple related constructs that may constitute “respect” to women.

With regard to the other two items that were significantly associated with the intervention, having the ASHA introduce herself might have stood out because this is a very easily obtainable, remembered and measured experience, and it was something that was specifically discussed in the PCFP training. Thus, it might have been more likely to have led to behavior change. This suggests that tangible examples of approaches to provide person-centered care that ASHAs could implement to improve experiences might be necessary to focus on and highlight in subsequent trainings. In summary, concrete behavior change points might be more amenable to intervention then vaguer concepts such as “showing the best care.”

One explanation for the lack of impact on a number of the items related to communication, choice of methods, and information is that these were covered in the training that ASHAs in both control and intervention areas received, as they related more to standard family planning counseling techniques and approaches. Thus, our added PCFP component might not have had an additional effect for intervention versus control ASHAs for these domains because these behaviors were addressed in both arms of the study. This suggests that our add-on training for PCFP could have focused more narrowly, which may have increased the impact on topics not covered in the other training. Unfortunately, we did not have the full, final standard training at the time of the development of our PCFP intervention and could not make these adjustments prior to the evaluation. The findings in the qualitative interviews suggested intervention ASHAs were aware of and seemed to practice domains of PCFP even prior to the intervention, suggests that this content was not new or unique to our intervention, adding support to this hypothesis.

The qualitative findings do suggest that, despite the lack of impact on actual PCFP scores, ASHAs who were part of the intervention clearly absorbed the material and felt that it led to changes in their thinking and behaviour. They also overwhelmingly liked the intervention and none mentioned feeling like it was material they already knew or did not need to know. This is promising in that it suggests that future, more focused interventions would likely be well received. However, it still begs the question of how to translate knowledge change into changes in behaviour.

We do find that women who saw intervention ASHAs were more likely to report that the provider had a strong or extremely strong preference for what method she choose. One of the main topics covered in the intervention was shared decision-making and the importance of not pressuring women to choose one specific method or another. However, it appears that women who saw intervention ASHAs felt that the ASHA had a strong preference, suggesting that this message was not incorporated into behaviour, and may have actually had the opposite effect. It is possible that women feeling pressured by the ASHA was an explanation for the lack of impact of the training on subsequent PCFP scores.

This study has numerous strengths, including matched control and intervention ASHAs, mixed methods, and an adequate sample size to test for significance. However, it also has several limitations, including those addressed above related to similarities between intervention and control training materials, and not having tested and validated the PCFP measure in a population of women seeking care for community health workers. To address the former, offering the PCFP training alone (not combined with the FP training ASHAs were already receiving) could clarify whether the PCFP content itself led to changes in ASHA behaviour and subsequent changes in women’s experiences and outcomes. To address the latter, as mentioned above, cognitive interviews with women seeing ASHAs about the items in the PCFP scale could help determine if these are more appropriate for this type of interaction. Additionally, we only tested this intervention in one urban part of Uttar Pradesh (Varanasi), therefore results are not generalizable to rural or other parts of Uttar Pradesh or India. Our rational for focusing in one city in one state was based in feasibility –this was where the standard family planning training program was being tested. Additionally, because this was a development and pilot study of the PCFP add-on, starting in a smaller population was appropriate (rather than an expensive, larger scale or multi-site study). It is possible that women living in rural areas have a different type of relationship with their ASHA as they are likely to know the ASHA better given small village sizes and thus, PCFP may be less or more important. Future work that aimed to test an improved and modified version of this intervention should consider testing it in rural populations as well as urban.

## Conclusion

Strengthening the case for the importance of person-centered care for family planning on family planning outcomes is important, and something we add to in this study. However, questions as to the best approach to do this, especially for community health workers, remain. We also provide evidence for the use of the PCFP scale among this different, often neglected, population of providers (community health workers). However, it is possible that other items are relevant in the context of home-centered care with community health workers who women most likely already know, and who provide other forms of care as well such as nutritional counseling. To conclude, community health workers are the first point of care for family planning provision in many countries and much more is needed to support this cadre, to help them provide high quality family planning care, and to understand how the nature of care provision differs between these and other health care providers.

## Supplementary Information

**Additional file 1.**

## Data Availability

The datasets used and/or analysed during the current study are available from the corresponding author on reasonable request.

## Abbreviations

PCC: Person-centered care
PCFP: Person-centered family planning
TCIHC: The Challenge Initiative for Health Cities
ASHA: Accredited Social Health Activist
